# Gastric Cancer With Peritoneal Metastasis—A Comprehensive Review of Current Intraperitoneal Treatment Modalities

**DOI:** 10.3389/fonc.2022.864647

**Published:** 2022-05-26

**Authors:** Aruna Prabhu, Deepti Mishra, Andreas Brandl, Yutaka Yonemura

**Affiliations:** ^1^Department of Surgical Oncology, Thangam Cancer Center, Namakkal, India; ^2^Digestive Unit, Champalimaud Foundation, Lisbon, Portugal; ^3^Department of Surgery, Campus Virchow-Klinikum, Charité-Universitätsmedizin Berlin, Berlin, Germany; ^4^Department of Regional Cancer therapy, Peritoneal Surface Malignancy Centee, Kishiwada Tokushukai Hospital, Kishiwada, Japan; ^5^Japanese/Asian School of Peritoneal Surface Oncology, Osaka, Japan; ^6^Department of Regional Cancer therapy, Peritoneal Surface Malignancy Center, Kusatsu General Hospital, Shiga, Japan

**Keywords:** gastric cancer, peritoneal metastases, intraperitoneal chemotherapy, cytoreductive surgery, HIPEC, PIPAC

## Abstract

The treatment of patients with peritoneal metastasis from gastric cancer continues to evolve. With various forms of intraperitoneal drug delivery available, it is now possible to reach the sites of peritoneal metastases, which were otherwise sub-optimally covered by systemic chemotherapy, owing to the blood peritoneal barrier. We conducted a narrative review based on an extensive literature research, highlighting the current available intraperitoneal treatment options, which resulted in improved survival in well-selected patients of peritoneally metastasized gastric cancer. Intraperitoneal chemotherapy showed promising results in four different treatment modalities: prophylactic, neoadjuvant, adjuvant, and palliative. It is now possible to choose the type of intraperitoneal treatment/s in combination with systemic treatment/s, depending on patients’ general condition and peritoneal disease burden, thus providing individualized treatment to these patients. Randomized controlled trials for the different treatment modalities were mainly conducted in Asia and lack further validation in the other parts of the world. Most recent application tools, such as pressurized intraperitoneal aerosol chemotherapy, seem promising and need to pass the ongoing clinical trials.

## 1 Introduction

Gastric cancer (GC) is the third leading cause of cancer-related deaths worldwide with peritoneal metastases (PM) from GC associated with poorer median survival, ranging from 4 to6 months (1–3). In last two decades, however, with the advent of cytoreductive surgery (CRS) and hyperthermic intraperitoneal chemotherapy (HIPEC), there is increasing evidence of improvement in survival in well-selected patients of peritoneally metastasized GC. Other than intraoperative HIPEC, normothermic intraperitoneal chemotherapy in the form of EPIC (early postoperative intraperitoneal chemotherapy), SIPC (sequential intraperitoneal chemotherapy), neoadjuvant systemic and intraperitoneal chemotherapy (NIPS), and pressurized intraperitoneal aerosolized chemotherapy (PIPAC) are the various ways in which the intraperitoneal route is being utilized for better drug delivery to the sites of PM, wherein the reach of systemic chemotherapy is known to be suboptimal, owing to the blood peritoneal barrier.

## 2 Treatment Modalities of Intraperitoneal Chemotherapy

Similar to the different types of application, evidence has been created for the respective treatment modalities, such as *prophylactic* for patients with absence but high risk for PM, *neoadjuvant*, and *adjuvant* for patients after complete CRS, and *palliative* over the past two decades. To shed more light on these various clinical indications, they will be separately listed and discussed in the following sections.

### 2.1 Prophylactic

Metachronus PM have been reported to occur in 15%–46% of patients with locally advanced GC even after a R0 resection and are the most common cause of death in these patients (2, 4). Even with advances in perioperative multimodality treatment regimens, the proportion of patients developing metachronus PM remains high. Risk factors for the development of metachronus PM are T3/4 tumors, lymph node positivity status, higher grade of tumor (grades 3/4), signet ring cell (SRC) histology, and diffuse infiltrative growth pattern. Several studies since 1994 (**Table 1**), including the meta-analysis by Xu et al., Yan et al., and Sun et al. have reported on the beneficial use of prophylactic HIPEC in these patients with higher risk of developing PM (5–7, 9–12, 15–17, 20).

**Table 1:** Studies on prophylactic IP chemotherapy on patients with locally advanced GC.

Year and Author	Study Design	No. of Patients	Study Group	Group/s Studied	Survival	Morbidity and Mortality
**HIPEC**						
1994 Hamazoe et al. (5)	RCT	82	Serosal invasion	Sx+ HIPEC (MMC 10 mg/ ml × 50–60 min) vs. Sx alone	5-year OS (NS): 64.3% vs. 52.5% Median OS: 77 m vs. 66 m	Morbidity (Leak): 4.8% vs. 7.5% Mortality: 0% vs. 0%
1994 Fujimura et al. (6)	RCT	58	Serosal invasion	Sx+ HIPEC (300-mg Cis + 30-mg MMC at 41°C–42°C × 60 min) vs. Sx + CNPP (at 37°C–38°C × 60 min) vs. Sx alone	1-, 2-, and 3-year OS: 95%, 89%, and 68% vs. 81%, 75%, and 51% vs. 43%, 23%, and 23%	Morbidity: 36.3% vs. 39.1% vs. NK Mortality: 0% vs. 0% vs. NK
1995 Ikeguchi et al. (7)	RCT	174	Serosal invasion	Sx + HIPEC (MMC, 80–100 mg/m^2^) vs. Sx alone	5-year OS – 51 vs. 46% (NS) (1–9 LNs positive: 66 vs. 44%)	Morbidity: 1.2% vs. 2.1%
1995 Takahashi et al. (8)	RCT	113	Serosal invasion	Sx + MMC CH (50-mg MMC) vs. Sx alone	3-year OS: 38 vs. 20% (p < 0.05)	Morbidity: 40.4% vs. 7.1% Mortality: 0% vs. 0%
1999 Fujimoto et al. (9)	RCT	141	Serosal invasion	Sx + HIPEC (MMC, 10 mg/ ml) vs. Sx alone	2-, 4-, and 8-year OS: 88%, 76%, and 62% vs. 77%, 58%, and 49% (p = 0.03)	Morbidity: 2.8% vs. 2.8% Mortality: 0% vs. 0%
2001 Kim et al. (10)	Pros Case-Control	103	Serosal invasion	Sx + HIPEC (MMC, 10 µg/ml × 120 min) vs. Sx alone	5-year OS: 32.7% vs. 27.1%	Morbidity: 36.5% vs. 33.3%
2001 Yonemura et al. (11)	RCT	139	T2-T4	Sx + HIPEC (30-mg MMC + 300-mg Cis at 42°C–43°C) vs. Sx + CNPP (at 37°C) vs. Sx alone	5-year OS 61 vs. 43 vs. 42%	Morbidity: 19 vs. 14 vs. 19% Mortality: 4 vs. 0 vs. 4%
2006 Zhu et al. (12)	Pros Case - Control	118	Serosal invasion	Sx + HIPEC (30-mg MMC + 300-mg Cis) vs. Sx alone	Mean OS: 61 vs. 43 m 2-, 4-, and 6 -year OS: 83%, 70.5%, and 67.9% vs. 63.7%, 52.1%, and 37.7%	Morbidity: 23.1% vs. 12.2% Mortality: 0% vs. 0%
2019 Yarema et al. (13)	Retro	37	Serosal invasion	Sx + HIPEC	Mean OS: 34m 1-year OS:	Morbidity: 29.1% Mortality: 5.1%
2019 Beeharry et al. (14)	RCT	80	Locally advanced cT3/4	Sx + HIPEC (Cis of 50 mg/m^2^; 60 min) vs. Sx alone	3-year DFS 93% vs. 65% (p = 0.0054)	Morbidity: 7.5% vs. 15% Mortality-: 0% vs. 0%
2004 Xu et al. (15)	Meta-analysis	1161: 11 studies	Locally advanced GC	Sx + IP chemotherapy in GC vs. Sx alone	Pooled Odds ratio: 0.51	–
2007 Yan et al. (16)	Meta-analysis	1,648: 13 studies	Locally advanced GC	Sx + IP chemotherapy in GC vs. Sx alone	HIPEC: HR, 0.60; HIPEC + EPIC: HR, 0.45	IP chemotherapy-Intra-abdominal abscess: HR, 2.37; Neutropenia: HR, 4.33
2012 Sun et al. (17)	Meta-analysis	1,062: 10 studies	Locally advanced GC	Sx + HIPEC vs. Sx alone	HIPEC with: MMC-RR, 0.75; 5FU-RR, 0.69; Overall RR, 0.73	BM suppression: RR, 1.68; Anastomotic leak: RR, 0.52; Bowel fistula: RR, 1.38, Adhesive ileus: RR, 0.79 Liver dysfunction: RR, 1.47 (all NS)
2016 Feingold et al. (18)	systematic review	2,029: 17 studies	locally advanced GC	Sx + HIPEC vs. Sx alone	HIPEC: 5-year OR 0.65 (p = 0.0015)	
2017 Desiderio et al. (19)	Meta-analysis	1,810:18 studies	advanced GC	Sx + HIPEC vs. Sx alone	HIPEC: 3-year OS RR 0.71 (p = 0.03) 5-year OS RR 0.82 (p = 0.01)	

Sx, Surgery; CNPP, continuous normothermic peritoneal perfusion; LNs, lymph nodes; CH, activated charcoal particles; BM, bone marrow; NS, not significant; RR, risk ratio; NK, not known; MMC, mitomycin C; Cis, cisplatin; 5FU, 5 fluro-uracil; OS, overall survival; DFS, disease-free survival.

The most recent data on use of prophylactic HIPEC has been reported by Yarema et al. and Beeharry et al. both in 2019. The study by Yarema et al. included 37 patients treated with radical surgery followed by prophylactic HIPEC (13). Out of the 37 patients, 29 had pT4a and eight had pT4b disease. The median OS was 34 months; 1-year OS was 91.7% and DFS was 82.3%. Level I evidence has been reported by Beeharry et al., who conducted a randomized controlled trial (RCT) on 80 consecutive patients of locally advanced GC (18. Patients were separated into two groups: prophylactic HIPEC group (Radical D2 gastrectomy + intraoperative HIPEC with cisplatin 50 mg/m^2^ for 60 min) and control group (Radical D2 gastrectomy only). The HIPEC group experienced a significantly better 3-year DFS (93% versus 65%, p = 0.005) and lower peritoneal recurrence rate (3% versus 23%, p < 0.05).

In a systematic review and random effect analysis of the role of adjuvant IP chemotherapy in resectable GC, reported by Feingold et al., maximal benefit was noted with intra-operative delivery and possibly with the use of Mitomycin C (MMC) (18). The meta-analysis by Desiderio et al. includes 1,810 patients with advanced GC [from nine RCTs and nine non randomized controlled trials (NRCTs)]; 731 undergoing gastrectomy + HIPEC and 1,079 undergoing standard gastrectomy alone, although no significant difference was noted in 1-year OS, the OS at 3 and 5 years did show a statistically significant difference favoring the HIPEC arm (RR 0.71, p = 0.03 and RR 0.82, p = 0.01) (19), which is in line with previous studies. In addition, HIPEC proved advantageous in preventing peritoneal recurrences (RR 0.63, p < 0.01). However, no benefit was reported in local, lymph nodal, liver, or other sites of distant recurrences.

#### 2.1.1 Ongoing Studies

The GASTRICHIP study (a prospective, open, RCT; NCT01882933) is currently accruing patients with resectable T3/4 GC with or without lymph nodal involvement and with or without positive peritoneal cytology at washing, treated with perioperative systemic chemotherapy and D1/D2 gastrectomy, to oxaliplatin HIPEC or not (21). The primary outcome is OS at 5 years with secondary outcome being RFS, morbidity, mortality, and quality of life.

The PREVENT trial (open-label, RCT; NCT04447352) including a total of 200 patients with localized and locally advanced diffuse or mixed type (Laurens’ classification) adenocarcinoma of the stomach and Type II/III GEJ (22). All included patients will receive three to six pre-operative cycles of docetaxel, oxaliplatin, leucovorin, and 5-fluorouracil (FLOT) and will be randomized 1:1 to receive surgery only and postoperative FLOT or surgery plus HIPEC (Cis 75 mg/m^2^ for 90 min) and postoperative FLOT. The primary endpoint is PFS/DFS.

### 2.2 Neoadjuvant

Studies focusing on the neoadjuvant, meaning IP use of chemotherapy before CRS, were mainly conducted in the eastern world using IP port systems. During the last years, evidence is growing in the western world, using mainly laparoscopic HIPEC, and most recently pressurized intraperitoneal chemotherapy (PIPAC) for chemotherapeutic administration. Studies using normothermic intraperitoneal chemotherapy (NIPEC) or HIPEC are illustrated in **Table 2**.

**Table 2:** Studies on neoadjuvant IP chemotherapy in patients with PM from GC.

Year and Author	Study Design	No. of Patients	Study Group	IP Treatment	Response Rate (%)	Median Overall Survival	Morbidity and Mortality
**NIPEC**							
2012 Fujiwara et al. (23)	Phase II	18	Cyto pos/PM	DOC: 40–60 mg/m^2^	62.5–78	24.6	
2013 Fushida et al. (24)	Phase II	27	PM	DOC: 35–50 mg/m^2^	22–51.9	16.2	
2013 Yamaguchi et al. (25)	Phase II	35	PM	PTX: 20 mg/m^2^	68–97	17.6	
2017 Ishigami et al. (26)	Phase II	100	Cyto pos/PM	PTX: 20 mg/m^2^	64	30.5	
2020 Yonemura et al. (27)	Pros case control	419	Cyto pos/PM	DOC: 30 mg/m^2^ CIS: 30 mg/m^2^	64.1	CC-0: 20.5 CC-1: 12.0	
**HIPEC**							
2017 Yonemura et al. (28)	Pros case control	53	PM	DOC: 30 mg/m^2^ CIS: 30 mg/m^2^	PCI regression	14.4m 19.2m	Morbidity: 22.2% Mortality: 3.7%
2021 Badgwell et al. (32)	Phase II	20	Cyto pos/PM	MMC: 30 mg CIS: 200 mg	n.a.	24.2 post CRS: 16.1	Grade III/IV: 25% Mortality: 0%

NIPEC, normothermic intraperitoneal chemotherapy; HIPEC, hyperthermic intraperitoneal chemotherapy; Cyto pos, positive cytology; PM, peritoneal metastasis; DOC, docetaxel; PTX, paclitaxel; CIS, cisplatin; MMC, mitomycin C; CC, completeness of cytoreduction; CRS, cytoreductive surgery; n.a., not available.

In 2006, the concept of neoadjuvant systemic and intraperitoneal chemotherapy (NIPS) was introduced by Yonemura et al. (29) NIPS comprises of oral S1 (tegafur/gimeracil/oteracil) of 60 mg/m^2^, from days 1 to 21, followed by 1-week rest. On days 1, 8, and 15 after the start of oral S1, cisplatin of 30 mg/m^2^, and docetaxel of 30 mg/m^2^ in 500 ml of saline are introduced intraperitoneally through an intraperitoneal (IP) port placed under local anaesthesia. Usuall8y, CRS and HIPEC is performed after five to six cycles of NIPS and 5 to 6 weeks after the last cycle of NIPS.

A new bidirectional intraperitoneal and systemic induction chemotherapy (BISIC) has been reported in 2014 by the same group, wherein 60 mg/m^2^ of oral S1 was administered on days 1 to 14 followed by 1-week rest. Cisplatin of 30 mg/m^2^ and docetaxel of 30 mg/m^2^ were administered by IP infusion, as in NIPS, on day 1, and docetaxel and cisplatin are then administered intravenously (IV) on day 8 (30). In 71.1% of patients, a positive cytology became negative after BISIC, and a complete cytoreduction was possible in 64% of the patients. Grades 3 and 4 morbidity were reported in 9% and 6.8% of patients with operative mortality of 4.5%. Patient selection is of utmost importance for gaining maximum benefit from these comprehensive treatment options.

The same group published long-term survival of patients with PM from GC, with the above multimodality treatment (27). Out of the 419 patients treated with NIPS/BISIC, a CC0 resection was possible in 266 (63.5%) with resultant 10-year survival of 8.3% and median OS of 20.5 months. They identified that Peritoneal Cancer Index (PCI) before NIPS ≤ 13, after NIPS ≤ 11, small bowel PCI ≤ 2, ≤ 5 involved peritoneal sectors, negative pre- and post-NIPS cytology, and complete cytoreduction were all associated with significantly favorable prognosis.

IP paclitaxel has also been evaluated in a prospective phase II study by Chia et al., in combination with systemic capecitabine and oxaliplatin (XELOX) in patients with GCPM (31). Forty-four patients were treated with IP paclitaxel (40 mg/m^2^ on days 1 and 8), intravenous oxaliplatin (100 mg/m^2^ on day 1), and oral Capecitabine (1,000 mg/m^2^ from days 1 to 14). Responders underwent CRS and HIPEC. On comparing with a retrospective historical cohort of 39 patients treated with systemic chemotherapy (SC) alone, the median OS for the IP and SC groups was 14.6 and 10.6 months, p = .002. The 1-year OS was 67.8% in the IP group and 32.3% in the SC group, p <0.001. The median PFS for the IP and SC group was 9.5 and 4.4 months, respectively, p <0.001.

After the initial experience of neoadjuvant laparoscopic hyperthermic intraperitoneal chemoperfusion (NLHIPEC) from Yonemura et al. (28), who showed a significant decrease in PCI from 14.8 ± 11.4 to 9.9 ± 11.3 (p < 0.0001) in patients with PM of GC, Badgwell et al. conducted a phase II trial using laparoscopic HIPEC with 200 mg of cisplatin and 30 mg of MMC in a neoadjuvant modality (32). Patients reached median overall survival rates of 16.1 months after CRS + HIPEC with a morbidity of 25% (grade III/IV) and mortality of 0% (**Table 2**).

### 2.3 Adjuvant

#### 2.3.1 Cytoreductive Surgery and HIPEC

After the first publication by Fujimoto et al. (4) in 1988, reporting on the successful use of hyperthermic chemotherapy in patients with GC with PM, there have been several reports confirming the benefit of CRS and HIPEC in well-selected patients of PM from GC (**Table 3**) (12, 36–42, 50, 51).

**Table 3:** Studies on adjuvant IP chemotherapy in patients with PM from GC.

Year and Author	Study Design	No. of Patients	Study Group	Disease Burden	CC0/1	Group/s Studied	Survival	Morbidity and Mortality
2011 Yang et al (33)	RCT	68	GC PM	Median PCI: 15	58.8%	CRS alone vs. CRS + HIPEC (120-mg Cis + 30-mg MMC)	Median OS: 6.5 m vs. 11 m 3-year OS: 5.9%	Morbidity: 11.7%
2014 Rudloff et al. (34)	RCT	9	GC PM	Mean PCI: 9.3	77.8%	CRS + HIPEC vs. systemic chemotherapy alone	Median OS: 11.3 m vs. 4.3 m	Morbidity: 77.8% Mortality: 11%
2021 Rau et al. (35)	RCT	105	GC PM	n.s.	n.s.	CRS alone vs. CRS + HIPEC (Cis of 75 mg/m^2^; MMC of 15mg/m^2^)	median OS 14.9m vs. 14.9m	Morbidity: 43.6% vs. 38.1% Mortality: n.s.
1996 Yonemura et al. (36)	Pros	83	GC PM	P1/2: 40 P3: 43	33.8%	CRS + HIPEC (30-mg MMC +500-mg Cis +150-mg etoposide) × 60 min	1-year OS: 43% 5-year OS: 11% **In CC0/1**-1-year OS: 61% 5-year OS: 17%	Morbidity: 7.2%
2004 Glehen et al. (37)	Pros	49	GC PM	Gilly Stage: I: 13 II: 5 III: 12 IV: 19	48.8%	CRS + HIPEC (MMC, 40–60 mg) × 90 min	Med OS: 10.3m **In CCR0/1** Med OS: 21.3 m 5-year OS: 16%	Morbidity: 27% 30-day Mortality: 4%
2004 Hall et al. (38)	Pros Case control	74	GC PM	Gilly Stage I–III: 5 vs. 29, Stage IV: 29 vs. 9	35.3% vs. 62.5%	CRS + HIPEC (40-mg MMC) × 120 min vs. Radical Sx	Median OS: 8 m vs. 7.8 m **HIPEC group**: Med OS: R0: 23.3 m, R1: 11.2 m, R2: 4.6 m	Morbidity: 35% vs. 17.5% 30-day Mortality: 0% vs. 15%
2005 Yonemura et al. (39)	Retro	107	GC PM	P1/2: 35 P3: 72	69% vs. 28%	CRS + HIPEC (30-mg MMC + 300-mg Cis + 150-mg Etoposide) × 60 min vs. Conv Sx + HIPEC	**For all patients**: Median OS: 11 m, 5-year OS: 6.7% **For CRS group**: Median OS: 19.2 m (CC0/1) vs. 7.8 m (CC2/3)	Morbidity: 43% vs. 8% Mortality: 7% vs. 0%
2006 Zhu et al. (12)	Pros Case control	22	GC PM	NK	NK	Sx + HIPEC (50-µg/ml Cis + 5 µg/ml MMC) × 60 min vs. Sx alone	Median OS: 10 m vs. 5 m	Morbidity: NK, Mortality: 0%
2008 Scaringi et al. (20)	Retro	26	GC PM	Gilly stage III–IV: 81%	30.8%	CRS + HIPEC (MMC of 120 mg MMC + Cis of 200 mg/m^2^) × 90–120 min	Median OS: 6.6m CC0: 15 m vs. ≥CC1: 3.9 m	Morbidity: 27% Mortality: 3.8%
2010 Glehen et al. (40)	Retro	159	GC PM	Mean PCI: 9.4	CC0: 56%, CC1: 25.2%	CRS + HIPEC ± EPIC (HIPEC- MMC of 30–50 mg/m^2^ ± Cis of 50–100 mg/m^2^ × 60–120 min OR Oxali of 360–460 mg/m^2^ ± Irino of 100–200 mg/m^2^ ± IV 5FU/LV × 30 min)	Median OS: 9.2 m, 1-, 3-, and 5-year OS: 43%, 18%, and 13% **CC0/1 group**: Median OS: 15 m, 1-, 3-, and 5-year OS: 61%, 30%, and 23%	Morbidity: 27.8% Mortality: 6.5%
2010 Yang et al. (41)	Pros	28	GC PM ± Ascitis	Median PCI: 12	CC0: 39.2% CC1: 21.4%	CRS + HIPEC (MMC 30 mg + Cis 120 mg) × 90–120 min	Estimated Med OS: CC0/1: 43.4 m CC2: 9.5 m CC3: 7.5 m	Morbidity: 14.3% Mortality: 0%
2013 Hultman et al (10)	Pros	18	GC PM (all treated with NACT)	Median PCI: 12 (8 patients)	CC0:75% CC1: 12.5%	CRS + HIPEC + EPIC (8 patients) (HIPEC - Cis of 50 mg/m^2^ + Doxo of 15 mg/m^2^ × 90 min OR Oxali of 460 mg/m^2^ + IV5FU/ LV of 500 mg/m^2^ × 30 min	Median OS: 14.3 m (8 patients) **CC0 patients**: Median OS: 19.1 m	Morbidity: 62.5% 90-day Mortality: 10%
2014 Magge et al. (42)	Pros	23	GC PM	Median PCI: 10.5	CC0/1: 95.7%	CRS + HIPEC (MMC of 40 mg) × 100 min	Median OS: 9.5 m 3-year OS: 18%	Morbidity: 52.2% Mortality: 4.3%
2016 Chia et al. (43)	Retro	81	GC PM	Median PCI: 6	100%	MMC or Cis or Oxali × 90 min	5-year OS: 18%	Morbidity: 44% Mortality: 6.2%
2018 Rihuete Caro et al. (44)	Retro	35	Cyto pos/GC PM	Median PCI: 8	94%	Cis: 100 mg/m^2^ Doxo: 15 mg/m^2^	Median OS: 16 m 3-year OS: 21.3%	Morbidity: 25.7% Mortality: 5.7%
2019 Yarema et al. (13)	Retro	70	GC PM	Mean: PCI 5.6	71.4%	MMC or Cis or Oxali or Doxo	Median OS: 12.6 m 3-year OS: 21.3%	Morbidity: 29.1% Mortality: 5.1%
2019 Rau et al. (45)	Retro	58	GC PM	Mean: PCI 8.3	79.3%	Cis: 75 mg/m^2^ MMC: 15 mg/m^2^	Median OS 9.8 m 3-year OS: 17.5%	Morbidity: 22.4% Mortality: 1.7%
2019 Bonnot et al. (46)	Retro	180	GC PM	Median PCI 6	CCO: 76.7% CC1: 23.3%	Various	Median OS: 18.4 m 3-year OS: 27.1%	Morbidity: 53.7% Mortality: 7.4%
2020 Rau et al. (47)	Retro	235	GC PM	Median PCI 8	CCO: 71.6%	Various	Median OS: 13 m 5-year OS: 6%	Morbidity: 17.0% Mortality: 5.1%

Pros, prospective study; Retro, retrospective study; Cis, cisplatin; MMC, mitomycin C; Oxali, oxaliplatin; Doxo, doxorubicin; 5FU, 5 fluro-uracil; LV, leucovorin; Sx, surgery.

Japanese staging system for PM (48).

P1: Peritoneal dissemination limited to the adjacent peritoneum of the stomach.

P2: Several scattered metastases in the distant peritoneum.

P3: Numerous metastases to the distant peritoneum.

Gilly’s staging system for PM (49).

Gilly stage 1: Malignant tumor nodules <5 mm in diameter, localized in one part of the abdomen.

Gilly stage II: Tumor nodules < 5 mm in diameter, diffuse to the whole abdomen.

Gilly stage III: Tumor nodules 5 mm to 2 cm in diameter.

Gilly stage IV: Large malignant nodules (>2 cm in diameter).

#### 2.3.2 PCI Threshold for CRS

Strict patient selection is of utmost importance, to ensure maximum benefit from these comprehensive treatment options. One of the important aspects in selection of patients for CRS and HIPEC is the disease burden. For patients with PM from GC, a PCI of maximum 10 to 12 has been suggested (52, 53). Even with complete CRS, benefit in OS is seldomly seen in patients with PCI > 12. Recent studies have suggested more stringent PCI cut offs; ≤ 6.

Chia et al. reported on 81 patients, from five French institutions who underwent CRS and HIPEC for PM from GC (43). Of the 81 patients, 59 had a complete cytoreduction with median PCI of 6 in these patients. The 5-year OS was 18% with nine patients disease free at 5 years (cure rate of 11%).

#### 2.3.3 Recent Literature

Recent data on the effectiveness of CRS and HIPEC on patients with GC exist from across the world with studies from high-volume centers and multicenter data pooling, along with RCTs and systematic reviews and meta-analysis.

There are data on CRS and HIPEC in patients from Central and Eastern European population by Yarema et al. (13). In all, 70 patients of PM from GC were treated with CRS and HIPEC at six of the Central and Eastern European HIPEC centers. The mean peritoneal carcinomatosis index (PCI) was 5.6. Complete cytoreduction was achieved in 71.4% of the patients. After CRS and HIPEC, 44 were treated with adjuvant systemic chemotherapy. The median OS was 12.6 months, and 1 year OS was 53.8%.

Despite most recent studies, it seems worthwhile mentioning the two largest studies from the western world, i.e., France and Germany. The CYTO-CHIP (Cytoreductive surgery versus Cytoreductive surgery and Hyperthermic Intraperitoneal Therapy) is an observational study of patients with GC with limited PM across 19 French treatment centers that were part of the BIG-RENAPE and/or the FREGAT groups (46). Patients with histologically proven PM and/or positive peritoneal cytology and/or ovarian metastases who had undergone CC0/1 were only included for the analysis. The inverse probability of treatment weighting (IPTW) approach was used to ensure that the two groups were similar in the observable characteristics. Except the median PCI that remained higher in the CRS-HIPEC group (6 versus 2, p= 0.003), the other parameters were balanced between the two study groups, after the IPTW adjustment. In total, 277 patients were included for the analysis; 180 underwent CRS and HIPEC, and 97 CRS alone. The median OS was 18.8 vs. 12.1 months in the CRS-HIPEC compared to the CRS alone groups, respectively; with 3- and 5-year OS rates being 26.2% and 19.9% versus 10.8% and 6.4% (adjusted HR, 0.60, p = 0.005), and 3-and 5-year recurrence-free survival (RFS) rates were 20.4% and 17.1% versus 5.9% and 3.8% (p = 0.001), respectively. No significant differences were noted between the two groups regarding the 90-day mortality (7.4% versus 10.1%, p = 0.820) or major complication rate (53.7% versus 55.3%, p = 0.496). The study results affirm the benefit of HIPEC in addition to CRS, in improving both OS and RFS in patients with limited PM from GC, without added morbidity.

Rau et al. reported on the effectiveness of CRS and HIPEC in 315 patients, of peritoneally metastasized GC, from the national German HIPEC registry initiated by the German Society of General and Visceral surgery (DGAV) (47). Patients with pathologically confirmed synchronous PM of GC from 2011 to 2016 were included in this analysis. Preoperative chemotherapy was used in majority of the patients (74%). A complete cytoreduction was possible in 121 patients (71.6%). The median OS was 13 months and 5-year OS was 6% for the entire study cohort. PCI was noted to significantly influence the median OS; PCI of 0–6: 18 months; PCI of 7–15: 12 months; and PCI of 16–39: 5 months (p = 0.002). This study stressed on the proper selection of patients with the use of staging laparoscopy for selecting patients for CRS and HIPEC.

Regarding long-term survival or even cure, an analysis by Brandl et al. shed more light on this topic in a multi-institutional cohort study from PSOGI including 28 patients (out of 448), with histologically proven PM of GC, treated with CRS and HIPEC, between 1994 and 2014 (54). The median OS was 11.0 years. The mean PCI was 3.3% and 78.6% of these patients had CC0 with PCI < 6. Thus, stating that long-term survival and even cure is possible in appropriately selected patients of PM from GC (54).

Most recently, the results of the GASTRIPEC trial, which was prematurely stopped due to slow recruitment, were published, in which a total of 105 patients were randomized to be treated either with CRS alone or CRS and HIPEC (35). The median OS for both groups was 14.9 months without any significant difference between both groups (14.9 versus 14.9 months; p = 0.165). While the treatment related morbidity was similar (grade >3 adverse events during NACT and 30 post-op days were similar in both groups; 46% and 43.6% in the CRS and HIPEC group, 62% and 38.1% in CRS alone group; p = 0.160 and p = 0.79, respectively), the PFS was significantly improved from 3.5 months (95% CI, 3.0–7.0) in the CRS alone group to 7.1 months (95% CI, 3.7–10.5; p = 0.047) in the CRS and HIPEC group (35).

#### 2.3.4 Ongoing Trials

The Dutch PERISCOPE II trial (NCT03348150) investigates the effect of CRS + HIPEC with oxaliplatin (460 mg/m^2^) for 30 min at 41°C–42°C, followed by docetaxel (50 mg/m^2^) for 90 min at 37°C in patients with limited PM (PCI < 7) compared to systemic chemotherapy (55). The inclusion of a total of 182 patients are intended; primary endpoint is 5-year overall survival.

### 2.4 Palliative

On the basis of the thesis of an improved efficacy using bidirectional chemotherapy (intravenously and intraperitoneally), several studies investigated the additional benefit on patient survival using IP chemotherapy in palliative indication, which are illustrated in **Table 4**.

**Table 4:** Studies on IP chemotherapy as palliative treatment in patients with PM from GC.

Year and Author	Study Design	No. of Patients	Study Group	Group/s Studied	Median Overall Survival	Morbidity and Mortality
**NIPEC**						
2013 Yamaguchi et al. (25)	Phase II	35	GC PM	IP + IV + S1	17.6 m	Morbidity: 34%
2018 Ishigami et al. (56)	RCT Phase III	164	GC PM	IP + IV + S1 IV + S1	17.7 m 15.2 m 3-year OS: 21.9% vs. 6.0%	Morbidity: 50% Mortality: 0%
**PIPAC**						
2017 Alyami et al (48)	Retro	73	GC PM	Cis: 7.5 mg/m^2^ Doxo: 1.5 mg/m^2^	Decreased PCI: 64.5%	Morbidity: 9.7% Mortality: 6.8%
2018 Khomyakov et al (49)	Phase II	31	GC PM	Cis: 7.5 mg/m^2^ Doxo: 1.5 mg/m^2^	13m major pathol. Response 60%	Morbidity: 0%
2019 Struller et al (57)	Phase II	25	GC PM	Cis: 7.5 mg/m^2^ Doxo: 1.5 mg/m^2^	6.7m pathol Response / Stable 40%	Morbidity: 0%
2020 di Giorgio et al (58)	Phase II	28	GC PM	Cis: 7.5 mg/m^2^ Doxo: 1.5mg/m^2^	12.3m pathol response 61.5%	Morbidity: 4% Mortality: 4%
2021 Alyami et al. (59)	Retro	42	GC PM	Cis: 7.5 mg/m^2^ Doxo: 1.5 mg/m^2^	19.1m	Morbidity: 6.1% Mortality: 4.7%

IP, intraperitoneal; IV, intravenously; S1, tegafur/gimeracil/oteracil; Retro, retrospective study; Cis, cisplatin; Doxo, doxorubicin; GC PM, gastric cancer peritoneal metastasis.

#### 2.4.1 Role of NIPEC

After the successful results of phase II (25, 60) studies, demonstrating efficacy and safety of IP paclitaxel, in 2018, Ishigami et al. reported on the first RCT, comparing combined IP paclitaxel and systemic chemotherapy with systemic chemotherapy in patients with PM from GC (56). The combination arm consisted of IP paclitaxel of 20 mg/m^2^ and IV paclitaxel of 50 mg/m^2^ on days 1 and 8 plus oral S1 of 80 mg/m^2^ daily from days 1 to 14 at 3 weekly intervals. The systemic chemotherapy arm consisted of daily oral S1 from days 1 to 21 with cisplatin of 60mg/m^2^ on day 8 at 5 weekly intervals. The treatment was continued, until disease progression, unacceptable toxicity, investigator decision or patient withdrawal. The median duration of treatment was 39 weeks in the IP arm and 15 weeks in the systemic chemotherapy arm. The median survival was 17.7 months in the IP arm versus 15.2 months in the systemic chemotherapy arm, not statistically significant (p = 0.080). However, after adjusting for baseline ascites, the HR was 0.59 (p = 0.008). The authors concluded that the efficacy of the IP regimen was underestimated by the primary analysis owing to the unexpected imbalance in the amount of ascites and the crossover from systemic to IP chemotherapy arms.

#### 2.4.2 Role of HIPEC

Control of malignant ascites can be achieved by HIPEC. Several reports along with a systematic review have shown ascites control in 95% of patients with the use of laparoscopic HIPEC (61–63). Recently, Yarema et al. reported on use of HIPEC to control malignant ascites in 10 patients. Mean volume of ascitic fluid was 5.5 liters ± 1.4 (3.5–8), and the mean PCI was 30.6 ± 6.1 (15–39). Although ascites elimination was achieved in all patients, giving symptomatic relief, this group, as expected, had poor median OS and DFS; 3.5months and 2.5 months respectively (13).

#### 2.4.3 Role of PIPAC

Pressurized intraperitoneal chemotherapy (PIPAC) using aerosolized system of drug delivery in the setting of capnoperitoneum has been increasingly used in the setting of unresectable PM and malignant ascites. Initial reports on the use of PIPAC in 24 patients of PM from GC, by Reymond et al., showed objective tumor response in 50% of the patients with PIPAC with 25% patients, having complete pathological response (64).

Alyami et al. reported on the use of PIPAC in 42 patients with unresectable PM, who were treated with PIPAC (cisplatin and doxorubicin) (59). The morbidity was low (6.1%), and a median overall survival of 19.1 months was reached.

Another study by Di Giorgio et al. reported on the safety and efficacy of PIPAC in 28 consecutive patients of GC PM from a single center, from September 2017 to September 2019 (58). Forty-six PIPAC procedures were performed with a mean of 1.7 PIPAC per patient. Pathological response was noted in 61.5% of patients (one with complete and seven with partial response). The median OS was 12.3 months for the entire cohort and 15 months in patients undergoing >1 PIPAC procedure (58).

Presently, there are several studies reporting on the safety, feasibility, and the effectiveness of PIPAC procedure with low-dose cisplatin (7.5 mg/m^2^) and doxorubicin (1.5 mg/m^2^) in patients with unresectable PM from GC (59, 65, 66). A systematic review by Garg et al. identified a total of 129 patients with GC PM treated with PIPAC (10 studies; two with an exclusive cohort of patients with GC and eight with a heterogeneous population with only a small proportion of GC patients). The review concluded that PIPAC is a safe and well-tolerated procedure with minimal peri-operative morbidity, with the potential to contain the spread of PM, at the same time improving or stabilizing the patients QoL (67).

#### 2.4.4 Ongoing Trials

Research on the further safety and efficacy of PIPAC procedure, drugs to be used, the optimal dose of drugs, etc., continue. The results of PIPAC EstoK 01—a prospective, open, randomized multicenter phase II study on patients with PM with GC, with PCI > 8—are awaited (68). Patients are being treated with either three cycles of PIPAC with oxaliplatin + systemic chemotherapy (one PIPAC then two IV chemotherapy) versus systemic chemotherapy alone. Two dose escalation studies on oxaliplatin PIPAC are also currently ongoing to determine the optimal dose to be used during PIPAC (69, 70). PIPAC GA 01 is yet another PIPAC trial on patients with recurrent GC, to evaluate the safety and efficacy of PIPAC with doxorubicin and cisplatin (three single doses in 6-week interval) (71).

## 3 Specific Subtypes

### 3.1 P0/Cy1

Patients with positive peritoneal fluid cytology without evidence of visible PM (P0/Cy1) need a special mention, because in spite of a curative resection, the median survival of these patients is similar to patients with obvious PM (14, 72). The AJCC (seventh edition) has also classified the presence of positive peritoneal cytology as M1 disease (73). These patients have been either treated with gastrectomy followed by adjuvant treatment (resulting in high rates of peritoneal recurrence) or with palliative intent chemotherapy. The effectiveness of neoadjuvant intraperitoneal chemotherapy on patients with positive peritoneal cytology has been demonstrated by studies on patients with PM and positive peritoneal cytology by Yonemura et al. (29, 30). These studies have reported positive cytology reverting to negative in 56% and 70% of the patients after neoadjuvant IP treatment, respectively. There are very few studies looking specifically at treatment of patients with only positive peritoneal cytology, as this factor is usually considered as an exclusion criteria.

In the study by Kuramoto et al., 88 patients of P0/Cy1 were randomized into three groups: surgery alone, surgery with IP chemotherapy, and surgery with extensive intraperitoneal lavage (EIPL) and IP chemotherapy (74). All patients were treated with adjuvant 5FU derivatives × 2 years. The 5-year OS was significantly higher in the surgery + EIPL+ IP chemotherapy group (44%) than in the surgery + IP chemotherapy (5%) and surgery alone group (0%). Similarly, the peritoneal recurrence was significantly lower in the EIPL group; 40%, 79%, and 90%, respectively. Thus, EIPL and IPC during surgery have shown beneficial effects in this group of patients. In another study, Imano et al. reported 100% conversion of positive cytology to negative with improved 5-year survival (5-year OS rate: 25%), for patients of P0/Cy1, treated with gastrectomy and EPIC using paclitaxel (75).

Ishigami et al. reported on the effectiveness of NIPS (IP and intravenous paclitaxel with oral S1), on patients with GC with PM or positive peritoneal cytology (26). Although the number of patients with only positive peritoneal cytology in their study was only 8, in comparison to the entire cohort of 100 patients, they did demonstrate improved median OS with this neoadjuvant treatment.

The recently reported CYTO-CHIP study by Bonnot et al. included 46 patients with PCI 0 (46). However, they also included patients with microscopic PM at the time of pathological examination or isolated ovarian Krukenberg tumors along with patients with positive peritoneal cytology as PCI 0. Of the 46 patients, 16 patients were treated with CRS-HIPEC and 30 with CRS alone. The median OS was 22.8 versus 12.9 months, respectively, a difference of 9.9 months, although not statistically significant due to small sample size.

In a review of various studies on patients of GC with P0/Cy1, Taniguchi et al. have concluded that postoperative oral S1, NIPS, or EPIC can result in cure in 25% to 44% patients by eradicating intraperitoneal micrometastasis (76).

Thus, these patients with only positive peritoneal cytology in the absence of obvious PM need to be identified by preoperative ascitic fluid or peritoneal wash cytology, so as to cater appropriate treatment, with the use of IP chemotherapy in some form along with CRS and HIPEC, to improve their prognosis.

### 3.2 Her2-Positive Gastric Cancer With PM

Her2 positivity has been identified in 13%–22% of all patients with GC (77, 78). In patients with PM from GC, the frequency of Her2 positivity has been found to be extremely low in the range of 2%–3% (79). Trastuzumab in combination with chemotherapy in patients with advanced gastric and gastro-esophageal cancers has shown survival advantage in this otherwise poor prognostic sub-group (80). Very few studies have been reported on the use of trastuzumab in patients with GCPM, considering the low frequency of Her2 positivity in this subgroup.

In 2014, Berretta et al., for the first time, reported on the use of IP Trastuzumab in a 61-year-old lady with pleural and peritoneal disease progression in a previously treated patient of advanced GC (81). The patient was initially treated with systemic chemotherapy with Trastuzumab along with weekly intra-pleural cisplatin, which resulted in complete pathological response at the pleural site of disease. IP Trastuzumab was then administered weekly at a dose of 150 mg for six cycles (after paracentesis). The patient had symptomatic relief without any local complications due to the IP Trastuzumab along with a stable peritoneal disease.

Recently, Li et al. reported on the use of α-emitting Trastuzumab in a mice model with PM of Her2 positive GC (82). Biodistribution analysis in the mouse model showed that IP administration of the α-emitting Trastuzumab was more uniform than IV administration and showed prolonged survival time as compared to the controls (two of six mice had complete response and three of six had good partial response).

### 3.3 Signet Ring Subtype of Gastric Cancer

SRC histology is known to be an aggressive subtype with poor prognosis. In comparison to appendicular and colorectal cancers, GC is more likely to have SRC subtype; 3.4% to 32.5% of all gastric cancers (83–85). The role of CRS and HIPEC in patients with PM from SRC GC is unclear.

In 2014, Konigsrainer et al. retrospectively analyzed 18 patients of SRC GC with synchronous PM treated with four to six cycles of neoadjuvant chemotherapy (5FU, folinic acid, docetaxel, and oxaliplatin) followed by CRS and HIPEC (cisplatin of 50 mg/m^2^ for 90 min at 42°C) (86). CC0/1 was achievable in 72% of patients. At a median follow-up of 6.6 months, the median OS was 8.9 months for patients with CC0/1, as opposed to 1.1 month for CC2/3. The PFS in patients with CC0/1 was 6.2 months. They concluded that prognosis of patients with PM from SRC GC remains poor, in spite of CRS and HIPEC and only a highly selected subgroup of patients after confirming response and resectability by a prior staging laparoscopy, should be subjected to this multimodality treatment to achieve any OS advantage.

Daniel et al. have reported on 204 patients with SRC histology from various primary gastrointestinal malignancies, treated with complete CRS followed by HIPEC from 2007 to 2016 (87). Of the 204 patients, 18 patients had primary GC. The median OS was 12 months for the patients with SRC GC, as compared to 27 months for SRC appendicular cancers and 18 months for the SRC colorectal cancers. Multivariate analysis of all 204 patients with SRC subtype showed GC origin to negatively influence survival (HR 4.59, p = 0.008) (87).

In the CYTO-CHIP study (previously mentioned), 188 of 277 patients had SRC (88). Median PCI was highest in the SRC-CRS + HIPEC group (median PCI of 7). The 3-year OS (after CRS ± HIPEC) was poor in the SRC group as compared to the non-SRC group (14% versus 38.4%, p < 0.001). However, within the SRC group, HIPEC was associated with better OS on multivariate analysis, than CRS alone (median OS 16.3 months versus 11 months, p = 0.003). They concluded that in well-selected patients of SRC GC with resectable PM, HIPEC is a valuable option.

Recent studies by Alyami et al. (59) and Bonnot et al. (65) on the use of PIPAC in patients with diffuse and unresectable PM from GC had significant number of patients with SRC histology; 33 of 42 patients and 79 of 91 patients, respectively. The median OS for the whole cohort was 19.1 and 15.1 months, respectively, thus indicating that PIPAC alternating with systemic chemotherapy may be the treatment of choice in future for this poor prognostic subgroup followed by reassessment for CRS and HIPEC in responding patients.

In addition, targeting tumor cells with loss of E-cadherin due to epithelial mesenchymal transition (EMT), which plays a central role in the loss of cohesiveness and increased chances of peritoneal dissemination in SRC cancers, is an interesting area of research in this subgroup of patients, which may eventually help improving their prognosis (89).

## 4 Discussion

The treatment armamentarium of patients with GC PM continues to expand. In these patients who had only systemic chemotherapy or best supportive care as their treatment options, in the past, they now can be treated with a wide variety of multimodality treatments.

With the advent of CRS and HIPEC, improved median OS has been reported, ranging from 11 to 23 months (13, 36–,12, 38, 44–47). The improvement in median OS is more pronounced in well-selected patients (good pre-operative functional reserve), absence of diffuse peritoneal involvement (PCI ≤ 12 or ≤ 6), absence of extraperitoneal metastasis, and when CC0/1 resection is possible. In a highly selected cohort study from PSOGI of 28 patients with >5-year OS, the median OS was reported to be 11.0 years (54). The mean PCI was 3.3% and 78.6% of these patients had CC0 resection with PCI < 6. Thus, in well-selected patients of PM from GC, even cure is a possibility.

When intraperitoneal chemotherapy is used in the neoadjuvant setting in conjunction with systemic chemotherapy (NIPS/BISIC), there is remarkable number of patients in whom CC0/1 resection may become feasible. This concept was first introduced by Yonemura et al. (29) and is now being widely used to downstage patients with diffuse peritoneal involvement, making them amenable to CRS and HIPEC. Several studies have shown significant decrease in PCI with combined IP/IV treatments, as well as conversion of ascitic fluid cytology from positive to negative with acceptable grade 3 and 4 morbidity and mortality (25, 27, 30, 31, 56, 60, 90). Various regimens are available and very well summarized by Brandl et al. with suggestions of regimens toward the latter part of the article with the intention to standardize these treatment protocols (53).

Metachronous development of PM occurs in 15%–45% of patients with locally advanced GC (T3/4 tumors, N2/3 lymph node positivity, high grade tumors, and SRC histology). Several studies, including systematic reviews and meta-analyses, have reported improved DFS and OS with prophylactic HIPEC (5–7, 9–12, 14–18, 91). The results of the GASTRICCHIP study, a prospective RCT on prophylactic HIPEC is eagerly awaited, before routine use of prophylactic HIPEC, across the world (21).

Patients with positive ascitic fluid cytology or peritoneal washings in the absence of obvious PM need to be treated aggressively with some form of IP chemotherapy, as we have studies demonstrating high chances of peritoneal recurrence when treated with surgery with or without systemic chemotherapy. Use of NIPS, EIPL, CRS and HIPEC, EPIC or post- operative prolonged S1 have shown to result in cure in 25% to 44% of patients by eradicating intraperitoneal micrometastasis (76).

Similarly, well-selected patients of the SRC histology (patients responding to neoadjuvant treatment, having limited PM, limited small bowel involvement) can have improved outcome with CRS and HIPEC. With the advent of PIPAC, even patients with diffuse peritoneal involvement may become amenable to complete CRS if good response to PIPAC alternating with systemic chemotherapy is noted in this otherwise poor prognostic subgroup (59, 65).

There is an overwhelming increase in data on the safety, feasibility, and efficacy of PIPAC in patients with diffuse PM from GC. In highly selected patients, initially deemed unresectable, a secondary CRS and HIPEC may become possible after repeated PIPAC cycles (92). Thus, patients who are not candidates for CC0/1 resection either upfront or after some form of neoadjuvant treatment may be considered for studies on PIPAC.

## 5 Conclusion

Thus, a favorable survival in patients with PM from GC has been seen with the various forms of IP chemotherapy. Proper patient selection in terms of patient fitness and peritoneal disease burden are key to maximize the benefit and minimize the morbidity and mortality from these available multimodality comprehensive treatment options. The importance of multidisciplinary team and treatment in high volume centers has also been time and again demonstrated to be of importance while treating patients with this aggressive disease. Further research in molecular subtypes of GC with multiplex profiling of PM from GC may eventually provide us with targets to provide more individualized treatment for these patients and thus result in favorable outcomes.

## Author Contributions

AP and DM: Data acquisition, manuscript preparation, and manuscript editing and review. AB and YY: Manuscript editing and review. All authors contributed to the article and approved the submitted version.

## Conflict of Interest

The authors declare that the research was conducted in the absence of any commercial or financial relationships that could be construed as a potential conflict of interest.

## Publisher’s Note

All claims expressed in this article are solely those of the authors and do not necessarily represent those of their affiliated organizations, or those of the publisher, the editors and the reviewers. Any product that may be evaluated in this article, or claim that may be made by its manufacturer, is not guaranteed or endorsed by the publisher.
